# Risk factors of postoperative recurrences in patients with clinical stage I NSCLC

**DOI:** 10.1186/1477-7819-12-10

**Published:** 2014-01-10

**Authors:** Ying-Yi Chen, Tsai-Wang Huang, Wen-Chiuan Tsai, Li-Fan Lin, Jian-Bo Cheng, Hung Chang, Shih-Chun Lee

**Affiliations:** 1 Division of Thoracic Surgery, Department of Surgery, Tri-Service General Hospital, National Defense Medical Center, Taipei, Taiwan; 2 Department of Pathology, Tri-Service General Hospital, National Defense Medical Center, Taipei, Taiwan; 3 Department of Nuclear Medicine, Tri-Service General Hospital, National Defense Medical Center, Taipei, Taiwan

**Keywords:** Non-small cell lung cancer, Locoregional recurrence, Distant metastasis, Carcinoembryonic antigen

## Abstract

**Background:**

Despite advances in radiation therapy, chemotherapy, and newly developed molecular targeting therapies, long-term survival after resection for patients with NSCLC remains less than 50%. We investigated factors predicting postoperative locoregional recurrences and distant metastases in patients with clinical stage I non-small-cell lung cancer (NSCLC) after surgical resection.

**Methods:**

All patients with clinical stage I NSCLC, who underwent surgical resection between January 2002 and June 2006, were reviewed retrospectively. Multiple logistic regression analyses were used to identify independent risk factors for patients with locoregional recurrences and distant metastases.

**Results:**

A total of 261 patients were eligible. Overall survival was significant related to locoregional recurrences (*P* = 0.03) and distant metastases (*P* <0.001). There were significant differences of locoregional recurrence in tumor differentiation (*P* = 0.032) and advanced pathological stage (*P* = 0.002). In the group of distant metastases, there were significant differences in tumor differentiation (*P* = 0.035), lymphovascular space invasion (*P* = 0.031). Among the relationship between pattern of distant metastasis and clinicopathologic variables in patients with clinical stage I NSCLC, SUVmax (*P* = 0.02) and tumor size (*P* = 0.001) had significant differences. According to multiple logistic regression analysis, tumor differentiation is the only risk factor of postoperative outcome for locoregional recurrence and serum CEA (>3.5 ng/mL) is the predictor of distant metastasis.

**Conclusions:**

Tumor differentiation and serum CEA were predictors of postoperative relapse for clinical stage I NSCLC after surgical resection. Risk factors of postoperative recurrence in patients with clinical stage I NSCLC may enable us to optimize the patient selection for postoperative adjuvant therapies or neoadjuvant treatment before surgery.

## Background

Non-small-cell lung cancer (NSCLC) remains the leading cause of cancer-related death and has a dismal prognosis. Despite advances in radiation therapy, chemotherapy, and newly developed molecular targeting therapies, long-term survival after resection for patients with NSCLC remains less than 50%. Most mortality following surgical resection is associated with tumor recurrence. Studies have investigated the clinical, pathological, and genetic factors that are associated with decreased survival after resection for NSCLC [1,2]. Nodal metastasis or occult micrometastasis might be a key-link of postoperative recurrence in patients with stage I NSCLC. Our study investigated factors predicting postoperatively locoregional recurrence or distant metastasis in patients with clinical stage I NSCLC after surgical resection. These clinicopathologic variables could be available before surgery and enable us to optimize the patient selection for postoperatively adjuvant therapies or preoperatively neoadjuvant treatment in order to reduce recurrences and improving survival rate.

## Methods

A retrospective examination was performed of a prospectively maintained database of patients who underwent surgical resection for clinical stage I NSCLC from January 2002 to June 2006 at Tri-Service General Hospital, Taiwan. The preoperative staging work-up included chest and upper abdomen computed tomography (CT) scans, bronchoscopic examination, nuclear medicine survey (bone and brain), and whole body positron emission tomography (PET) scan. Mediastinoscopy was not a routine preoperative staging procedure, and was performed only when enlarged mediastinal lymph nodes (diameter >1.0 cm) shown by the CT scan to prove nodal status of staging. All patients underwent surgical intervention for cure. Patients were excluded if they had carcinoid histological type, second lung primary lesions, or died within 30 days of operation. The selected cases were staged according to the seventh edition of the American Joint Committee on Cancer adopted in 2009 [3]. After evaluation of the resectability and operability of the tumors, a total of 261 patients with clinical stage I NSCLC were enrolled and underwent surgical resection with dissection of the mediastinal lymph nodes. Serum carcinoembryonic antigen (CEA) level was measured as a part of the routine preoperative evaluation and postoperative follow-up. Serum CEA level was calculated by means of the two-site immunoenzymometric assay (CEA test; CIS Bio International, France; reference range <5.0 ng/mL) following the manufacturer’s instructions. The upper limit of normal in our hospital defined as 3.5 ng/mL based on the 95% specificity level for benign lung disease.

Postoperatively, patients were initially seen at 2 to 3 weeks after resection by the thoracic surgeon and then again every 3 to 6 months; they underwent contrast-enhanced chest CT at these appointments during the follow-up period. The serum CEA level was routinely measured after surgery every 3 months. Subsequent to these visits, either a roentgenogram or CT scan of the chest was reviewed annually. PET with CT or magnetic resonance imaging was used as clinically warranted. All visits were completed in concert with the referring oncologist. Recurrence was documented either radiographically or histologically in all cases.

Second primary lung cancer was differentiated from recurrent NSCLC according to the criteria proposed by Martini [4]. Clinicopathologic variables were investigated for their influence on time to locoregional recurrences and distant metastasis. For evaluation of surgical prognosis, patients with locoregional recurrences or distant metastases were independently analyzed and discussed. Locoregional recurrence was defined as tumor recurrence in contiguous anatomical sites, including the ipsilateral hemithorax and ipsilateral mediastinal or hilar lymph nodes, or both after surgical resection. Distant metastasis was defined as tumor recurrence in the contralateral lung or outside the hemithorax and mediastinum after surgical resection.

### Statistical analysis

Descriptive data are expressed as the mean ± standard deviation. Student’s *t* test was used to investigate continuous variables and the χ^2^ test was used to compare categorical variables between these groups. Survival from the date of surgery was calculated using Kaplan-Meier survival analysis. Multiple logistic regression analyses were used to identify independent risk factors for patients with locoregional recurrences and distant metastases. SPSS 14.0 software (SPSS, Inc., Chicago, IL, USA) was used for all analyses and statistical significance was defined as *P* <0.05.

## Results

Two hundred sixty-one patients with clinical stage I NSCLC after complete resection and dissection of mediastinal lymph nodes were reviewed. In our study, 17 patients (6.5%) of the total of 261 patients had locoregional recurrences and 20 (7.66%) of the same population had distant metastases. The most common site of locoregional recurrence is ipsilateral lung (10/17, 58.82%) and that of distant metastasis are brain (9/20, 45%) and bone (8/20, 40%). Three of 17 patients with locoregional recurrence had pleural disseminations and the other four patients had mediastinal lymph node recurrences. Two of 20 patients with distant metastasis had liver metastases and only one patient had adrenal gland metastasis.

**Figure 1:**
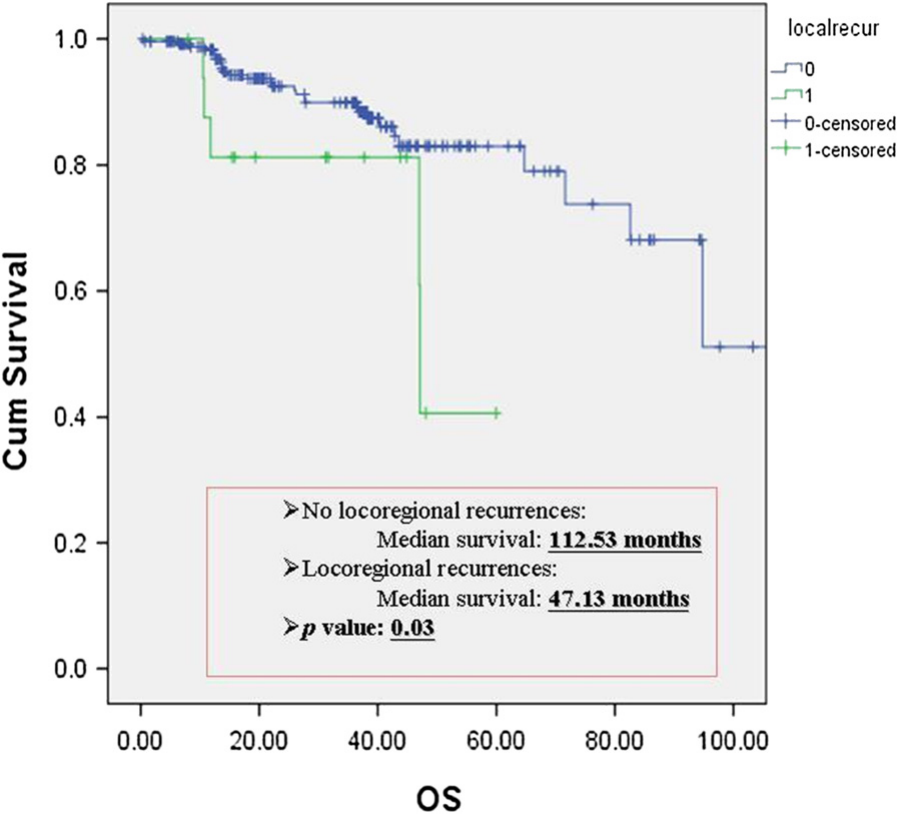
**The relationship of overall survival rate between with and without locoregional recurrences in clinical stage I NSCLC.** Cum, cumulative; OS, overall survival.

**Figure 2:**
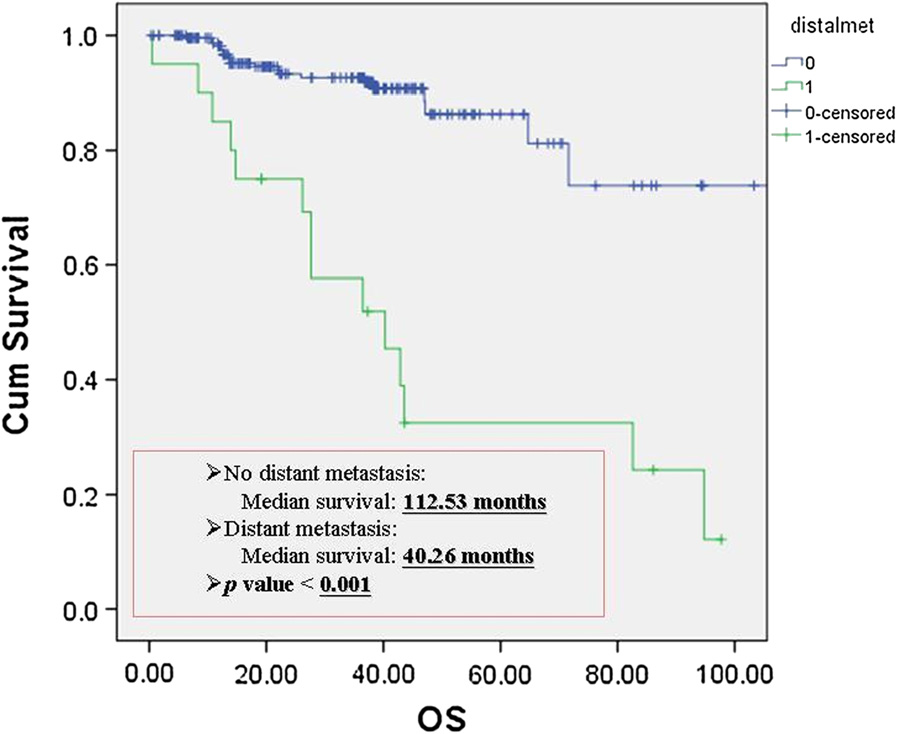
**The relationship of overall survival rate between with and without distant metastasis in clinical stage I NSCLC.** Cum, cumulative; OS, overall survival.

Figure 1 reveals the relationship between cumulative survival rate and overall survival in patients with locoregional recurrence. The median survival showed 112.53 months in patients without recurrence *versus* 47.13 months in patients with recurrence (*P* = 0.03). Figure 2 showed the relationship between cumulative survival rate and overall survival in patients with distant metastasis. The median survival showed 112.53 months in patients without metastasis *versus* 40.26 months in patients with recurrences (*P* <0.001). Therefore, surgical outcome of these patients is associated with postoperative recurrences.

The demographic characteristics in patients with clinical stage I NSCLC are shown in Table 1. Recurrences are subdivided into locoregional recurrences and distant metastases. There were no significant differences in sex (*P* = 0.806; *P* = 0.989), tumor histopathology (*P* = 0.14, *P* =0.322), tumor location (*P* = 0.324; *P* =0.494), and different surgical procedures (*P* = 0.609; *P* =0.620). Comparisons between two groups showed statistical significance only in tumor differentiation (*P* = 0.032; *P* = 0.035). Seventy-five patients (75/244, 30.74%) in the group without locoregional recurrence had postoperative upstaging *versus* seven patients (7/17, 41.18%) with recurrence (*P* = 0.002). There were 20 distant metastases, representing 7.66% of the population studied. These data without significant differences in locoregional recurrence and with significant difference in distant metastasis were administration of adjuvant chemotherapy (*P* = 0.44; *P* <0.001), lymphovascular space invasion (LVSI) (*P* = 0.214; *P* =0.031).

**Table 1:** Characteristics of patients with or without loco-regional recurrence and distant metastasis after resection for clinical stage I NSCLC

	**Locoregional recurrence**			**Distant metastasis**		
	**No (*n*** **= 244) (100%)**	**Yes (*n*** **= 17) (100%)**	***p*-value**^**a**^	**No (*n*** **= 241) (100%)**	**Yes (*n*** **= 20) (100%)**	***p*-value**^**a**^
Sex:						
**Male**	**110 (45.08)**	**7 (41.18)**	**0.806**	**108 (44.81)**	**9 (45)**	**0.989**
**Female**	**134 (54.92)**	**10 (58.82)**		**133 (55.19)**	**11 (55)**	
Histopathology:						
**Adenocarcinoma**	**209 (85.66)**	**17 (100)**	**0.140**	**210 (87.14)**	**16 (80)**	**0.322**
**Other**	**35 (14.34)**	**0 (0)**		**31 (12.86)**	**4 (20)**	
Differentiation:						
**Well**	**98 (40.16)**	**4 (23.53)**		**99 (41.08)**	**3 (15)**	
**Moderate**	**97 (39.75)**	**5 (29.41)**	**0.032**	**93 (38.59)**	**9 (45)**	**0.035**
**Poor**	**49 (20.08)**	**8 (47.06)**		**49 (20.33)**	**8 (40)**	
Location:						
**Central**	**125 (51.23)**	**11 (64.71)**	**0.324**	**124 (51.45)**	**12 (60)**	**0.494**
**Peripheral**	**119 (48.77)**	**6 (35.29)**		**117 (48.55)**	**8 (40)**	
Adjuvant chemotherapy:						
**Yes**	**89 (36.48)**	**8 (47.06)**	**0.440**	**80 (33.2)**	**3 (15)**	**< 0.001**
**No**	**155 (63.52)**	**9 (52.94)**		**161 (66.8)**	**17 (85)**	
LVSI:						
**Absent**	**222 (90.98)**	**14 (82.35)**	**0.214**	**221 (91.7)**	**15 (75)**	**0.031**
**Present**	**22 (9.02)**	**3 (17.65)**		**20 (8.3)**	**5 (25)**	
Operation:						
**Lobectomy**	**228 (93.44)**	**17 (100)**	**0.609**	**225 (93.36)**	**20 (100)**	**0.620**
**Wedge**	**16 (6.56)**	**0 (0)**		**16 (6.64)**	**0 (0)**	
p-stage:						
**I**	**169 (69.26)**	**10 (58.82)**	**0.002**	**168 (69.71)**	**11 (55)**	**0.232**
**II**	**44 (18.03)**	**3 (17.65)**		**40 (16.60)**	**7 (35)**	
**III**	**31 (12.71)**	**3 (17.65)**		**32 (13.28)**	**2 (10)**	
**IV**	**0 (0)**	**1 (5.88)**		**1 (0.41)**	**0 (0)**	

^a^Significance was assessed using χ^2^ tests. Key: SCC, squamous cell carcinoma; LVSI, lymphovascular space invasion; p-stage, pathology stage.

Statistically significant *p* values are depicted in underline.

Table 2 illustrates the association of clinicopathologic variables to predict the locoregional recurrence or distant metastasis. These factors included age, preoperative SUVmax of tumor, tumor size, and serum CEA level. Despite of no significant difference in SUVmax of tumor and CEA level, patients with locoregional recurrence still have higher value than patients without recurrence. Those in the distant metastasis group had a higher SUVmax (4.44 ± 3.96 *vs*. 6.69 ± 4.39, *P* = 0.020) and larger tumor size (2.67 ± 1.49 cm *vs*. 3.83 ± 1.41 cm, *P =* 0.001). The preoperative CEA levels (5.01 ± 12.65 ng/mL *vs*. 10.31 ± 23.34 ng/mL, *P* = 0.346) was not significantly associated with distant metastasis, which was obviously more than that without distant metastasis.

**Table 2:** Relationship between pattern of locoregional recurrence and distant metastasis with clinicopathologic variables in patients of clinical stage-I NSCLC

***Variables***	**Locoregional recurrence**			**Distant metastasis**		
	**No (n = 244)**	**Yes (n = 17)**	***p*-value**^**a**^	**No (n = 241)**	**Yes (n = 20)**	***p*-value**^**a**^
**Age (y)**	**61.51 ± 12.32**	**59.94 ± 10.14**	**0.609**	**61.11 ± 12.33**	**65.00 ± 9.82**	**0.170**
**SUVmax of tumor**	**4.55 ± 4.05**	**5.79 ± 3.91**	**0.251**	**4.44 ± 3.96**	**6.69 ± 4.39**	**0.020**
**Tumor size (cm)**	**2.76 ± 1.54**	**2.76 ± 1.07**	**0.991**	**2.67 ± 1.49**	**3.83 ± 1.41**	**0.001**
**CEA (ng/mL)**	**4.59 ± 9.29**	**14.58 ± 33.44**	**0.267**	**5.01 ± 12.65**	**10.31 ± 23.34**	**0.346**

^a^Significance was assessed using Student’s *t* tests; SUVmax: maximum standard uptake value; CEA: carcinoembryonic antigen.

Statistically significant *p* values are depicted in underline.

**Figure 3:**
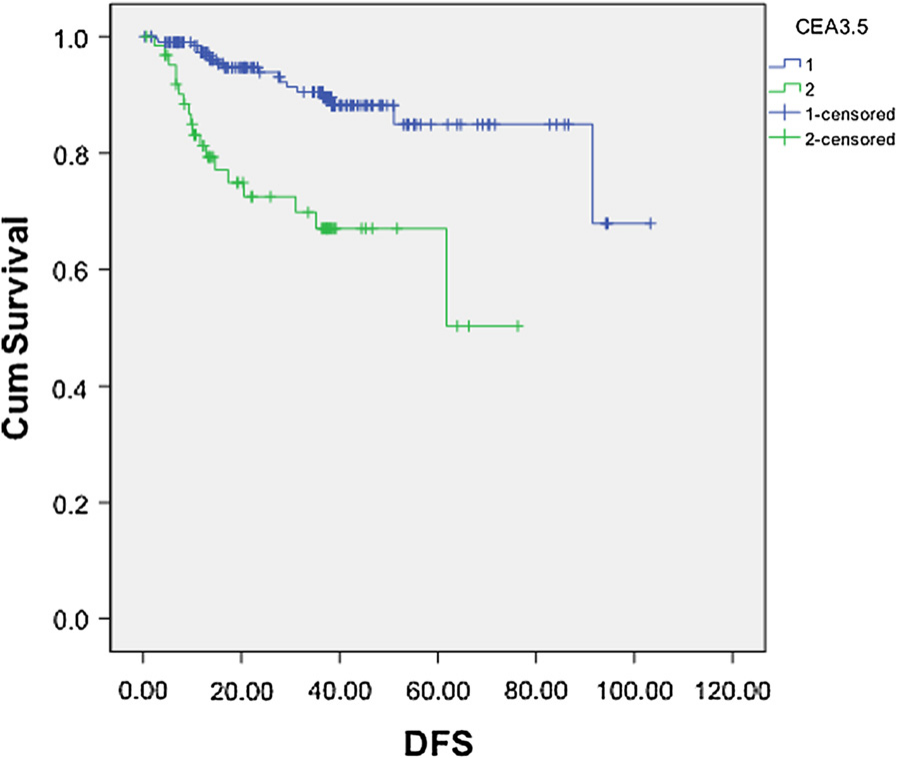
**The relationship of DFS and CEA level in clinical stage I NSCLC.** The CEA level can predict DFS after operation (the blue line: CEA <3.5 ng/mL; the green line: CEA >3.5 ng/mL; *P* <0.001). CEA, carcinoembryonic antigen; cum, cumulative; DFS, disease-free survival.

In the multiple logistic regression analysis (Table 3), poor tumor differentiation (odds ratio (OR): 4.902, *P* = 0.007) was the only independent risk factor associated with postoperatively locoregional recurrence. Preoperative SUVmax ≥3.3 (OR: 1.193, *P* = 0.766), CEA ≥3.5 ng/mL (OR: 2.545, *P* = 0.110), tumor size >2 cm (OR: 1.243, *P* = 0.721), and LVSI (OR: 2.103, *P* = 0.336) did not predict postoperatively locoregional recurrence. The only independent predictor of postoperatively distant metastasis was preoperative serum CEA ≥3.5 ng/mL (OR: 3.505, *P* = 0.029). In the Figure 3, the CEA level can also predict disease-free survival after operation (*P* <0.001).

**Table 3:** Multiple logistic regression analysis for locoregional recurrences and distant metastases in patients with clinical stage-I NSCLC

**Factor**	**Locoregional recurrence**		**Distant metastasis**	
	**OR (95% CI)**	***p*-value**	**OR (95% CI)**	***p*-value**
SUVmax ^3^ 3.3	1.193 (0.373–3.818)	0.766	1.430 (0.470–4.735)	0.608
CEA ^3^ 3.5 ng/mL	2.545 (0.810–8.001)	0.110	**3.505 (1.138–10.795)**	**0.029**
Tumor size > 2 cm	1.243 (0.377–4.098)	0.721	3.349 (0.668–16.782)	0.142
Differentiation	**4.902 (1.541–15.625)**	**0.007**	1.615 (0.186–2.062)	0.434
LVSI	2.103 (0.462–9.573)	0.336	1.430 (0.365–5.601)	0.608

Key: OR, odds ratio; CI, confidence interval. Differentiation: non-poor/poor.

Statistically significant *p* values are depicted in bold print.

## Discussion

Lung cancer has the highest incidence and mortality rates of any major cancer worldwide. The 5-year survival rate has been reported as 73% for patients with pathology stage IA and 58% for pathological stage IB [5]. After apparent complete resection in patients with stage I disease, the recurrence rates range from 25% to 50% [6]. In our study, we utilize the same population to probe into surgical outcome on the occurrence of locoregional recurrence or distant metastasis. Figures 1 and 2 showed significant differences between overall survival and postoperative recurrences. The survival rate is strongly associated with locoregional recurrence or distant metastasis. The mechanism of postoperative recurrence was not well established to date. Godfrey et al. [7] stated that one possible reason for this may be that those patients with a poor outcome actually have more extensive disease, with occult locoregional and/or distant metastasis than originally identified by routine pathologic staging techniques. One of the most convinced etiology of postoperative recurrence was occult tumor cell spreading to lymph nodes or bone marrow in patients with NSCLC [7]. Chakrabarti et al. [8] thought the detection of bone marrow micrometastasis changes the staging and management of lung cancer, especially in NSCLC, where treatment with curative intent is planned, which can be suitably done by morphological study of bone marrow aspirate and biopsy. However, the cost effect of bone marrow aspiration is high and the invasiveness of the procedure may produce more complications. Therefore, we searched clinicopathologic factors, which were cost-effective and available before surgery, to predict postoperative recurrences in patients with clinical stage I NSCLC. Then, we could optimize the patient selection for neoadjuvant systemic therapy or postoperatively adjuvant treatment to reduce recurrent rate and improve survival rate.

In our data of locoregional recurrences, poor prognostic factors were poor tumor differentiation and advanced pathology stage (Table 1). Furthermore, no parameters were identified as independent risk factors (Table 2). Besides, the poor prognostic variables in patients with clinical stage I NSCLC who developed distant metastasis after surgical resection included poor tumor differentiation, absence of adjuvant chemotherapy, and advanced pathologic staging (Table 1). In the adjusted association of clinicopathologic variables with distant metastasis, the independent risk factors were SUVmax of tumor and tumor size (Table 2). In the multiple logistic regression analysis (Table 3), poor tumor differentiation (OR: 4.902, *P* = 0.007) was the only independent risk factor associated with postoperatively locoregional recurrence. The only independent predictor of postoperatively distant metastasis was preoperative serum CEA ≥3.5 ng/mL (OR: 3.505, *P* = 0.029). We would like to discuss these variables in the following subject matter.

Preoperative clinical staging is a key factor influencing the decision on the initial therapy for patients with lung cancer. In this regard, PET-CT with fluorodeoxyglucose (^18^F) uptake (FDG) plays an established role in the treatment of patients with NSCLC. FDG uptake, which reflects the tumor’s glucose metabolic rate, varies widely and depends on the histopathology type and aggressiveness of the tumor [9,10]. Consideration of this parameter enhances the accuracy of clinical staging, improving patient selection for surgical treatment. Several studies have reported that the preoperative SUVmax has prognostic value in patients with early stages of NSCLC [11,12]. Cerfolio et al. [13] reported a large series of 315 patients with resected NSCLC. In that study, a high SUVmax correlated with stage, recurrence, and survival. SUVmax ≥10 was the best independent predictor of disease-free survival and overall survival. Koo et al. [14] investigated factors associated with recurrence in 310 patients with stage I or II disease. There were 106 recurrences in the study population and a SUVmax ≥4.5 was found to be an independent predictor of recurrence after resection, with an OR of 5.45. The study did not stratify patients based on the location of recurrence to determine whether SUVmax was more predictive for locoregional than for distant recurrences. In another study, subgroup analysis found that a SUVmax >5 was associated with distant recurrences, whereas the predictive nature of SUVmax >5 did not reach statistical significance among patients with locoregional recurrences [15]. Although the metabolic activity of tumors has been shown to contribute significant information in terms of prognosis [16,17], the cutoff values for SUV measurements vary widely, making their clinical application difficult. In our study, the cutoff point of SUVmax of 3.3 was used according to our previous study [18]. However, the SUVmax of our results did not show statistical significance in prediction of locoregional recurrences and distant metastases.

There was concordance between the pathological stage and the preoperative clinical stage in 68.58% of patients in our both groups. Therefore, 82 patients (31.42%) had advanced disease upon the proof of pathologic stage. This was higher than the rates of upstaging tumors of 14.3% to 17% reported in the literature [19,20]. Varlotto et al. investigated factors associated with recurrence in 373 patients with stages I through IIIA NSCLC who underwent complete resection. An advanced pathologic stage was associated with an increased risk of distant recurrence [21]. Although the study established the importance of the pathological stage for the risk of recurrence, they used the sixth edition not the most recent seventh edition of the TNM staging system. Pepek et al. [22] compared the ability of the seventh edition TNM staging systems to detect locoregional recurrence in comparison to the sixth edition. Converting from the system used in the sixth edition to that in the seventh edition resulted in a 21% migration in stage classification (13% upstaged and 8% downstaged). This might explain the high rate of postoperative upstaging in our study, because we used the seventh edition of the TNM staging system for all patients. In our study, the rate of locoregional recurrence was 6.51% (3.83% for pathological stage I and 2.68% for non-stage I). A pathologic stage greater than I was independently associated with a higher risk of recurrence. Tumor differentiation, absence of adjuvant chemotherapy, and LVSI significantly associated with distant metastasis for clinical stage I NSCLC. But, the locoregional recurrences were only significantly related with tumor differentiation. The patients, who received adjuvant chemotherapy, got significantly lower rates of distant metastasis and better survival rates. Therefore, adjuvant chemotherapy may need to be considered, even for pathological stage I NSCLC patients. A prospective randomized study will be needed to clarify this issue.

Sublobar resection has been found to be an independent predictor of locoregional recurrence [15]. Tumor size in that report was not associated with an increased risk of recurrence in the sublobar resection group (2.3 cm in the group with recurrence, 2.2 cm in the group without recurrence; *P* <0.3). In contrast, Bando et al. [23] reported higher recurrent rate is associated with sublobar resection in patients with tumors >2 cm. In their study, patients with tumors <2 cm had locoregional recurrence rates of 1.9% compared with 33% in patients with tumors >2 cm. The different conclusions in these studies might be associated with the larger sample size in the former study and the fact that only 50% of the patients underwent wedge resection. In contrast, all patients underwent segmentectomy in the latter study. In our present study, only 16 patients had wedge resection and others underwent lobectomy. For patients with tumor diameters <2 cm, the postoperative recurrence rate was 6.1% *versus* 19.4% for patients with tumors >2 cm. After adjustment for the other factors, tumor size with a cutoff of 2 cm was not found to be an independent predictive factor in the multiple logistic regression analysis for patients of NSCLC.

Furthermore, histopathology markers were found to be prognostic predictors in patients with NSCLC [24]. CEA is a well-known tumor marker for substantial malignant tumors, including NSCLC. Evaluating serum CEA level is useful for monitoring response to chemotherapy and predicting relapse of advanced NSCLC [25]. Sawabata et al. [26] assessed 297 consecutive patients with clinical stage I NSCLC for evaluation of CEA level and the upper limit of normal defined as 7.0 ng/mL. They saw the CEA level as a useful predictor of survival for patients with clinical stage I NSCLC, and a persistently high CEA level after surgery as an especially strong indicator of a very poor prognosis. In another study, Buccheri et al. [27] reported the frequency of abnormal serum concentrations of CEA is low (17%), but it is important to identify such a small group of high-risk patients as many of them (55% and 70% of those with a CEA value in excess of 5 and 10 ng/mL, respectively; normal reference values <5 ng/mL) will develop an early postoperative recurrence. Despite of significant difference in serum CEA level in both groups of our study, CEA levels (≥3.5 ng/mL) was only an independent predictive factor for postoperative distant metastasis after adjustment of the other factors. The CEA level can also predict disease-free survival after operation. Then after an apparently successful operation, patients with clinical stage I NSCLC should be carefully followed up by intensified imaging study or close intervals. These patients could represent a suitable target for neoadjuvant clinical trials of selected high-risk groups. Apparently, they also received benefits from adjuvant chemotherapy with better survival rates in our study.

From literature review, Subotic et al. [28] stated that the intensified follow up did not increase either the proportion of patients detected with asymptomatic relapse or the number of patients with specific oncological treatment of relapse. However, the above follow-up method was regular monthly telephone contact with patients and/or their families in order to get reliable information about the patient’s general condition and eventual new complaints that were not present on discharge. We did not think the above follow-up method was a good way of early detection according to patient’s symptoms, because recurrent malignancy would be asymptomatic. The follow-up methods and intervals were more important for early detection of the recurrence. In our study, regular follow-up with imaging study and routinely measuring serum CEA level would be a better method for detection of small or occult recurrent lesions.

As a retrospective, single-center study, patient-selection bias and time-trend bias were inevitable. It was difficult to obtain performance status from the charts. Although performance status is clearly an important factor in stage IV NSCLC, its importance in stage I disease has not yet been established. Lack of data about performance status might have little significance in the analysis of death and recurrence in our study. The 261 patients were operated on by different surgeons. As we know, different surgeons have different levels of dexterity, leading to different rates of recurrence. Furthermore, the fact that the predictors demonstrated in our study were not all consistent with other reports highlights the possibility of false positive findings. More data are needed with a larger sample of patients and longer follow-up. Further studies of clinical information combined with histopathological markers might provide valuable indicators for recurrences. Prospective multi-institutional studies are mandatory to validate the predictors of recurrence in clinical stage I NSCLC.

## Conclusion

Tumor differentiation and serum CEA were predicators of postoperative relapse for clinical stage I NSCLC after surgical resection. Risk factors of postoperative recurrence in patients with clinical stage I NSCLC may enable us to optimize the patient selection for postoperative adjuvant therapies or neoadjuvant treatment before surgery.

### Consent

All patients who were included in our study signed the informed consents. This study has been proved by Institutional Review Board in our hospital.

## Abbreviations

CEA: Carcinoembryonic antigen; EUS-FNA: Esophageal ultrasound fine-needle aspiration; FDG: Fluorodeoxyglucose (^18^ F); LVSI: Lymphovascular space invasion; NSCLC: Non-small-cell lung cancer; PET-CT: Positron emission tomography–computed tomography; SUVmax: Maximum standard uptake value.

## Competing interests

There was no substantial direct or indirect commercial financial incentive associated with publishing this article.

## Authors’ contributions

Y-YC reviewed related literatures, participated in the sequence alignment and drafted the manuscript. T-WH collected patients’ data and performed the statistical analysis, and drafted the manuscript. W-CT reviewed patients’ surgical specimens and was responsible for pathohistological diagnosis in all patients of our study. L-FL reviewed the data of SUVmax in our patients. J-BC participated in revision of the manuscript. HC participated in revision of the manuscript and drafted the manuscript. S-CL conceived of the study, participated in its design and coordination. All authors read and approved the final manuscript.
